# Tackle your Tics, a brief intensive group-based exposure treatment for young people with tics: results of a randomised controlled trial

**DOI:** 10.1007/s00787-024-02410-0

**Published:** 2024-04-04

**Authors:** A. P. Heijerman-Holtgrefe, C. Huyser, M. Bus, L. P. L. Beljaars, J. M. T. M. van de Griendt, C. W. J. Verdellen, K. J. Kan, B. J. H. Zijlstra, R. J. L. Lindauer, D. C. Cath, P. J. Hoekstra, E. M. W. J. Utens

**Affiliations:** 1 Academic Center for Child and Adolescent Psychiatry, Levvel, Amsterdam, The Netherlands; 2 Department of Child and Adolescent Psychiatry, Amsterdam UMC Location University of Amsterdam, Amsterdam, The Netherlands; 3 Dutch Tourette Association, Haarlem, The Netherlands; 4 Expertisecentrum Valora, Veldhoven, The Netherlands; 5 TicXperts, Heteren, The Netherlands; 6 PsyQ Nijmegen/Parnassia Group, Nijmegen, The Netherlands; 7 Research Institute of Child Development and Education, University of Amsterdam, Amsterdam, The Netherlands; 8 Department of Specialized Training, GGZ Drenthe, Assen, The Netherlands; 9 Department of Psychiatry, University Medical Center Groningen, University of Groningen, Groningen, The Netherlands; 10 Department of Child and Adolescent Psychiatry and Accare Child Study Center, University Medical Center Groningen, University of Groningen, Groningen, The Netherlands; 11 Department of Child and Adolescent Psychiatry/Psychology, Erasmus MC – Sophia Children’s Hospital, Rotterdam, The Netherlands; 12 Department of Emotional Disorders, Levvel, Meibergdreef 5, Amsterdam, The Netherlands

**Keywords:** Tourette syndrome, Chronic tic disorder, Exposure and response prevention, Behavioural treatment, Quality of life

## Abstract

Tics can have a serious impact on the quality of life of children and their families. Behavioural therapy is an evidence-based first line treatment for tic disorders. This randomised controlled trial studied the efficacy of a brief, condensed group-based programme for children with tics (Dutch Trial Registry NL8052, 27 September 2019). Tackle your Tics is a four-day group treatment, including exposure and response prevention and supporting components, delivered by therapists and ‘experts by experience’. We collected outcome measures at baseline (T1), directly post-treatment (T2), and at three- and 6-months follow-up (T3, T4) including tic severity (primary outcome measure), tic-related impairment, quality of life, tic-related cognitions, emotional/behavioural functioning, family functioning, treatment satisfaction and adherence. Outcomes directly post-treatment improved in both the treatment group (n = 52) and waiting list (n = 54), but showed no statistically significant differences between the conditions (differential change over time T1-T2) on tic severity (Yale Global Tic Severity Scale), quality of life (Gilles de la Tourette Syndrome Quality of Life Scale), tic-related cognitions and family functioning. At longer term (T3), again no between-group difference was found on tic severity, but tic-related impairment, quality of life and emotional/behavioural functioning significantly improved in the treatment group compared to the waiting list. Mean treatment satisfaction scores were favourable for both children and parents. Directly posttreatment, Tackle your Tics showed no superior effect compared to waiting list. However, on longer term this brief four-day group treatment was effective in improving tic-related impairment, quality of life and emotional/behavioural functioning.

**Supplementary Information:**

The online version contains supplementary material available at 10.1007/s00787-024-02410-0.

## Introduction

Tics and their co-occurring problems can have major consequences for the daily life of children and their families. Tourette Syndrome (TS)^1^ is defined as the presence of both multiple motor tics and one or more vocal tics (not necessarily concurrently), during more than one year, starting before the age of 18 years, and not attributable to a substance or another medical condition [1]. This condition is associated with lower quality of life [2, 3] and daily functioning [4]. Also parents of children with TS experience a lower quality of life and worse family functioning than parents of typically developing children [5].

Behavioural therapy is recommended as first-line treatment for tic disorders in international clinical guidelines [6, 7]. Meta-analyses reported medium to large effect sizes on reducing tic severity [8]. Next to the well-established Comprehensive Behavioural Intervention for Tics (CBIT; standardised mean difference (SMD) = – 1.43) and habit reversal therapy (HRT; SMD = – 0.93), evidence has also been found for exposure and response prevention (ERP; SMD = – 1.37) [9]. While CBIT and HRT focus primarily on the most bothersome tics, ERP aims to suppress all tics at the same time, which may be an advantage in treating children with multiple tics, or in group treatment [10].

Innovative treatment modalities, using new formats, have been developed to optimise outcomes of behavioural treatment. Group formats have multiple advantages in the treatment of tics and other problems [11], such as reduced waiting lists, peer support, opportunities for ‘normalisation’, generalisation of the learned skills in social situations, and stimulation of motivation and homework adherence, a predictive factor for favourable treatment outcome [12]. Group therapy based on HRT [13, 14] and CBIT [10] showed improvements in tic severity, quality of life and comorbid symptoms [15]. Nissen and colleagues compared combined HRT and ERP in a group setting with individual treatment and found no significant differences in reductions of total tic scores [16]. Some studies, however, suggest a possible absent or negative effect of group treatment on vocal tics [10, 13]. Another innovative format is condensing treatment; offering an equal number of therapy hours within a shorter period of time reduces travel hours and treatment duration, with potentially faster results and less dropout. Two case series have suggested that brief, condensed treatments with CBIT [17] and ERP [18] are equally effective as regular 12-week therapies with one-hour sessions. Another innovation is to broaden treatment outcomes by combining behavioural therapy with supporting components, such as coping strategies, to deal with tic-related problems and improve quality of life (e.g. ‘Living with tics’ [19].

Considering the above, we developed Tackle your Tics, providing ERP to address multiple tics at once, while using a brief, condensed treatment in group format in a four-day period. As recommended by patient representatives, experts by experience (young adults with tics) offered coping strategies for tic-related issues in daily life [20]. Moreover, parent meetings and parent–child ERP-sessions were included. A previous pilot study into Tackle your Tics demonstrated its feasibility, positive treatment satisfaction and promising outcomes on tic severity and quality of life [21].

This study’s primary aim was to investigate whether Tackle your Tics led to superior reduction on the primary outcome of tic severity compared to a waiting list condition, with an effect size of at least 0.5. We also investigated effects on the secondary outcomes of tic-related impairment, quality of life, tic-related cognitions, emotional/behavioural functioning, family functioning, treatment satisfaction and adherence; and aimed to identify possible predictors of treatment outcome.

For these aims we conducted an investigator-blinded randomised controlled trial. We hypothesised that participation in the Tackle your Tics treatment would improve tic severity and secondary outcomes of children and parents on short and long term.

## Methods

A detailed description of the methods, including procedures, intervention and measurement instruments, was described in our study protocol [22]. A comprehensive summary is included below.

### Trial design

This investigator-blinded, randomised controlled trial studied the efficacy of the Tackle your Tics treatment (TYT), compared with a waiting list control group (WLCG, 3 months waiting) in children and adolescents with tic disorders in a 1:1 ratio.

### Ethical considerations

The study was registered at the International Clinical Trials Registry Platform (NL8052), approved by the medical ethical committee of Amsterdam UMC (NL66340.018.18) and adhered to the Declaration of Helsinki. Written informed consent was received from all parents and participants over 12 years. Representatives of the Dutch patient organisation continuously reviewed the research process, as members of the research team [23].

### Participants

Children and adolescents and their parents were recruited from July 2020 to May 2022 by the Dutch Tourette Association and three participating Dutch paediatric expert centres on tic disorders: Levvel, Yulius and Accare.

Children and adolescents had to meet the following inclusion criteria to be eligible for participation: (a) age 9–17 years, (b) having been diagnosed with TS or another chronic tic disorder (CTD), in accordance with the diagnostic criteria of the Diagnostic and Statistical Manual of Mental Disorders, 5th edition [1], (c) with at least moderate tic severity as indicated by a total tic score of more than 13 (or more than 9 for participants with motor or vocal tics only) on the Yale Global Tic Severity Scale, (YGTSS; [24]).

Exclusion criteria for participation were: (a) having received behavioural treatment for tics in the past 12 months (to safeguard that children were motivated to relearn the ERP exercises), (b) receiving pharmacological treatment for tics that has not been stable for the past six weeks or with planned changes during study participation, (c) poor mastery of the Dutch language, (d) IQ < 75, (e) serious physical disease, (f) substance abuse, (g) suicidality, (h) psychotic disorders, (i) poor group functioning or low motivation, as reported by the child, parents or local clinician. Co-occurring attention-deficit/hyperactivity disorder (ADHD), obsessive–compulsive disorder, anxiety disorders or mood disorders were no reason for exclusion, unless immediate treatment or an adaptation to the treatment protocol was required.

Demographics and medical history were derived from a clinical intake interview. Families reported the child’s age, sex and gender, cultural background, family composition, marital status, child’s education, parent’ highest completed educational level, parents’ professions, monthly family income (1–6 scale), previously diagnosed comorbidities, previous or current treatment for tics or other psychiatric problems and family history of tics. Parents’ professional level was determined by the coding system ISCO-08, 1–4 scale (International Standard Classification of Occupations: ISCO-08 [25].

Psychiatric comorbidities were determined with an online semi-structured interview at baseline (Anxiety Disorder Interview Schedule; ADIS, parent and child version) [26]. To limit unnecessary burden, the child version was only completed with children aged 12 years or older, who were able to give a one-hour online interview independently.

### Interventions

Participants in the TYT condition received treatment, in groups of 4–8 participants within 1 month after randomisation. Tackle your Tics is a brief, condensed group treatment with evidence-based ERP [27]. In this treatment, participants are trained to suppress all their tics simultaneously for a prolonged time (response prevention) while focussing on tolerating the preceding sensations or ‘tic alarms’. Treatment sessions were supplemented with innovative, supportive components including daily, one-hour workshops about living and coping with tics given by experts by experience. These young adult patients, trained for this study, taught the children how to cope with their symptoms in a positive, creative way. In the workshops, three themes were discussed and visualized (by writing, painting and mind mapping): self-acceptance, solution-oriented thinking and positive characteristics and strengths. See Appendix 1 for an overview of the programme content. Due to COVID-19 regulations, the treatment programme was slightly modified by providing all parent meetings online instead of face-to-face and introducing basic safety COVID-19 measures, in accordance with national guidelines, such as 1.5-m distance and self-tests. Online participation was available if a participant would not be able to complete the treatment face-to-face due to quarantine. Treatment was provided by therapists with 3–15 years of experience in treating tic disorders, assisted by co-therapists. Depending on group size (4–8 participants), a team of 2–3 experienced therapist and 1–2 co-therapists, and 1–2 trained experts by experience provided the programme. Treatment took place at Levvel, an academic centre for child and adolescent psychiatry in Amsterdam, the Netherlands.

Treatment integrity was enhanced by team intervision meetings before each treatment group and assessed by two independent, trained raters (bachelor students and one expert psychologist). Standardised forms to score the required programme components were used to rate a random 20% of all sessions, based on observations or audio recordings (forms available upon request).

Participants in the WLCG received this same treatment after a waiting period of 3 months, in which they received no psychosocial treatments for tics.

### Outcomes

Our primary outcome was tic severity, as assessed by the blinded, independent researcher (AH) in online interviews with the children, accompanied by and – if necessary – helped by their parents. Tic severity was measured by the total tic score of the YGTSS, covering the last week. This semi-structured interview for the assessment of tic severity is used in most clinical trials as the primary outcome measure [28] and has good reliability and other psychometric properties [29], as well as high reliability between online and in-person ratings [30]. A revised version of the YGTSS (YGTSS-R, [31]) was translated and analysed for this study as sensitivity analysis.

As secondary outcome measures, we used the YGTSS motor and vocal tic scores and the YGTSS tic-related impairment rating, which is an additional question to assess overall impairment in daily functioning that the child experiences as a consequence of having tics. Quality of life was measured by the Gilles de la Tourette Syndrome Quality of Life Scale for children and adolescents; C&A-GTS-QOL, total problem score and life satisfaction score [32]. We rated patients’ beliefs and cognitions about their tics using the Beliefs About Tics Scale [33]. This 20 item self report scale measures tic-related cognitions as beliefs about the sense of relief after ticking or the (negative) experiences while suppressing tics. The higher the score, the higher the degree to which the subject negatively perceives his/her experience when suppressing tics (or resisting tic urges).

As additional secondary outcome measures, tic-related cognitions, emotional/behavioural functioning, family functioning (including parenting stress, care-related quality of life in informal caregivers and overall family functioning) and treatment satisfaction and adherence were assessed. On the treatment satisfaction surveys, mean scores higher than 3 on a 5-point scale indicated a favourable treatment satisfaction. For the instruments used and their psychometric characteristics, see Table 1. All secondary outcomes apart from the YGTSS tic-related impairment rating and Outcome and Session Rating Scales (ORS/SRS) were completed online.

**Table 1:** Used Instruments, their Psychometric Characteristics and Time points

Variable	Questionnaire	Items	Score range	Score indication	T1	T2	T3
Primary outcome							
Tic severity^a^	Yale Global Tic Severity Scale (YGTSS)	11	0–100	tic severity	R	R	R
Secondary outcomes							
Quality of life	Gilles de la Tourette Syndrome Quality of Life Scale for children and adolescents (C&A-GTS-QOL)	27	27–135,	degree of problems in daily life and life satisfaction (0–100)	C	C	C
Tic-related cognitions	Beliefs about Tics Scale (BATS) (Dutch translation)	20	20–80	2degree of tic-related cognitions	C	C	
Emotional/behavioural functioning	Child Behaviour Checklist (CBCL6-18)	112	0–220	edegree of emotional and behavioural problems	P		P
Quality of life related to health	EQ-5D / EQ-5D-Y	5	1–5 per item	health related quality of life	C,P	C,P	
Stress of parenting	Stress of parenting questionnaire (OBVL)	34	34–136	stress of parenting	P	P	
Care-related quality of life in informal caregivers (parents)	Care Related Quality of Life (CarerQoL)	7	0–100	burden of providing informal care	P	P	
Family functioning^a^	Family Assessment Device (general functioning subscale)	12	12–48	degree of problems in family)	P	P	
Treatment satisfaction/homework adherence	Treatment satisfaction forms, developed for this study (child/parent version)	17 (C) 28 (P)	11–55 22–110 (5-point-scale items)	treatment satisfaction		C,P	C,P
Therapeutic alliance, patient functioning	Outcome Rating Scales and Session Rating scales (during treatment, child/youth version)	8	0–40 per scale	treatment satisfaction			
Predictor variables							
Demographic data	Sex, gender, age				R		
Psychiatric comorbidities	Anxiety Disorders Interview Schedule			presence of comorbidities	C, P		
Premonitory urges	Premonitory Urges for Tics Scale	9	9–36	tic-related feelings and sensations (premonitory urges)	C*		

*P* parent report, *C* child report, *T* teacher report, *R* researcher/clinician report

^a^Tic severity and family functioning at baseline are also included in the predictor analyses (see Methods and Analysis)

**Fig. 1:**
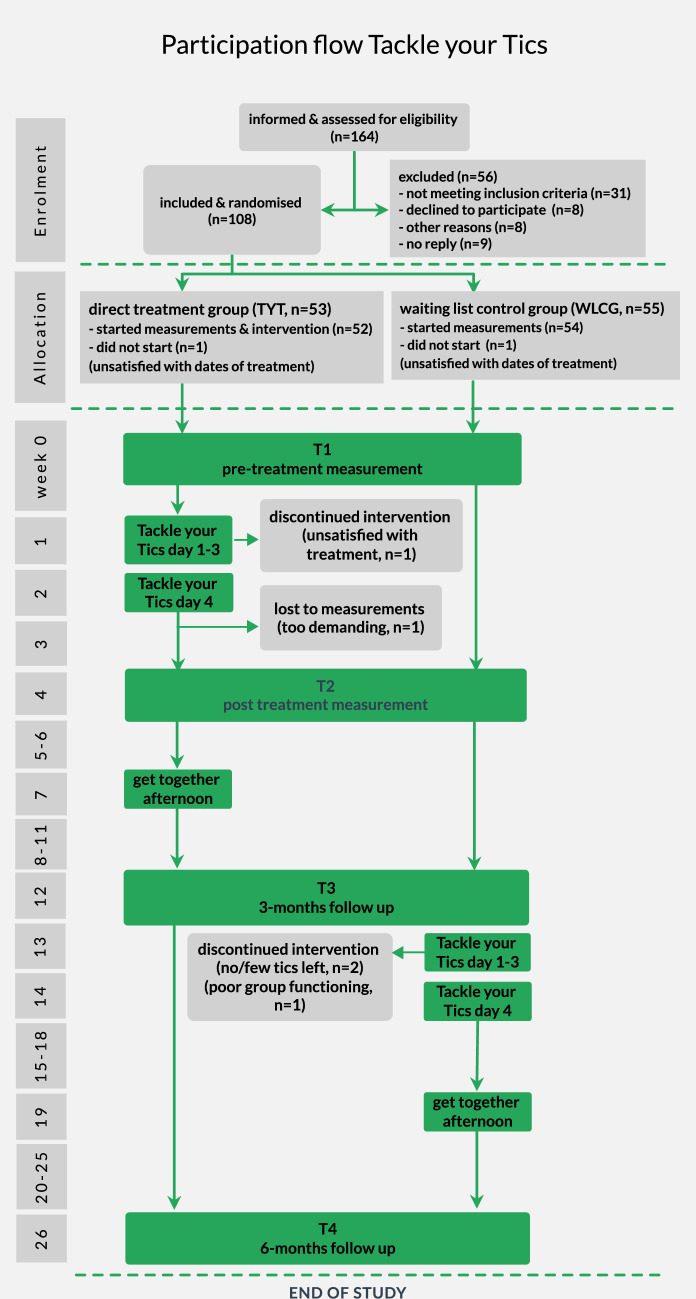
Participation Flow of the Tackle your Tics Study

All outcomes were assessed at baseline (T1; for TYT: within two weeks before the start of the treatment; for WLCG: at the start of the study), post-treatment (T2, for TYT: one week after the fourth treatment day; for WLCG: four weeks after T1), at 3-months (T3, both conditions: 3 months after T1) and 6-months follow-up (T4, both conditions: 6 months after T1) at parallel time intervals for participants in the TYT and WLCG condition (see Fig. 1).

### Sample size

A sample size of 104 participants was needed to detect univariate differences between TYT and WLCG (*p* ≤ 0.05) at the primary outcome on T2, at an effect size of Cohen’s d = 0.5, with a power of 0.80.

### Randomisation

A research assistant randomly assigned eligible participants using a computerised data management system (Castor EDC) to either direct treatment (TYT) or the waiting list (WLCG), using block randomisation (block size 2–4 participants) and stratification by gender.

### Blinding

Included families were informed of randomisation outcome prior to the baseline assessments, since they had to make practical arrangements (e.g., taking time off from work, making hotel reservations). However, the investigator who assessed the primary outcome measure was blinded to treatment allocation.

### Statistical analyses

SPSS Version 28 was used for all analyses (IBM SPSS Statistics for Windows, NY: IBM Corp). Results with p < 0.05 were considered statistically significant. For significant results, the standardised mean difference (SMD) was calculated as a measure of effect size, considering values of 0.2–0.5 as small, values of 0.5–0.8 as medium, and values > 0.8 as large effect sizes.

Differences in background characteristics between participants and non-participants (who did not meet the inclusion criteria or did not provide consent) as well as differences in baseline characteristics between TYT and WLCG were analysed by independent t-tests, Chi-square tests or Fisher’s exact tests.

To determine the efficacy of Tackle your Tics, difference in change on the primary outcome YGTSS total tic severity scores over time (T1–T2 and T1–T2–T3) between the TYT condition versus WLCG were compared with Generalised Estimating Equations (GEE) [34], which accounts for the dependency between repeated observations. This way, we examined the interaction between time and group as a test of effectiveness. Since the WLCG received treatment after T3, analyses were conducted from T1 to T3. T4-results were reported descriptively. Participants were included in the analysis if a score was available at one or more assessments.

In the same way, difference in change in secondary outcomes between the TYT and WLCG were tested with GEE. For each outcome, a separate GEE analysis was conducted.

As sensitivity analyses, we reanalysed our data to check for possible influence of deviations from the protocol, i.e. (1) missed programme components (> 3 h of children’s sessions or > 2 h of parent meetings), (2) a different treatment than planned (> 3 h online participation or a different condition than randomised) or (3) changes in medication. Deviations were included as covariates in the GEE-analysis, to investigate if these had obscured the original analyses.

Furthermore, we examined whether tic severity at baseline, premonitory urge severity, age, sex, family functioning, and comorbidity predicted treatment response. These outcomes were included as covariates in the GEE-analyses, to test if these led to an extra differential change over time.

To compare the number of children with a positive treatment response in both treatment groups by means of a Chi square test, a 25% reduction of the total tic score of the YGTSS from pre- to post-treatment was defined as a positive response [29].

## Results

### Participant flow

The participation flow is displayed in Fig. 1. After assessing 164 families for eligibility, we included 108 children. See Appendix 2 for more details.

### Baseline data

Table 2 shows the participant characteristics at baseline. Participants in TYT and WLCG did not significantly differ from each other at baseline as to demographic and medical characteristics.

**Table 2:** Baseline Participant Characteristics for total sample, direct treatment and waiting list condition

Characteristics	Total (N = 106)	TYT condition (n = 52)	WLCG (n = 54)	p value
Participant’s age, M ± SD	12.59 ± 2.11	12.60 ± 2.09	12.59 ± 2.14	0.977^a^
Sex, n (%)				
Male	71 (67%)	35 (67.3%)	36 (66.7%)	1.000^c^
Gender, n (%)				
Male	73 (68.9%)	37 (71.2%)	36 (66.7%)	0.834^c^
Cultural background, n (%)				
Dutch/European	96 (90.6%)	46 (88.5%)	50 (92.6%)	0.496^c^
Family composition, n (%)				
Multi-parent family	81 (76.4%)	39 (75.0%)	42 (77.8%)	0.888^c^
Divorce of biological parents, n (%)				
Not divorced	84 (80.8%)	41 (80.4%)	43 (81.1%)	0.924^b^
Patient’s education, n (%)				
Regular education	83 (78.3%)	38 (73.1%)	45 (83.3%)	0.554^c^
Parent’s highest completed education level, n (%)				
High (Bachelor’s/ Master’s degree)	78 (75.0%)	38 (74.5%)	40 (75.5%)	0.909^c^
Parent’s highest professional level, n (%) (ISCO-08 levels 1–4)				
High (level 4; corresponding with high educational level)	66 (63.5%)	32 (64.0%)	34 (63.0%)	0.994^b^
Monthly family income, n (%) (1–6 scale)				
High (level 6; > 5000 euro net)	29 (34.1%)	13 (31.7%)	16 (36.4%)	0.862^a^
Tic severity at baseline, M ± SD (YGTSS-interview at T1)				
Motor tic score	17.02 ± 4.28	16.27 ± 4.30	17.74 ± 4.18	0.077^a^
Vocal tic score	11.21 ± 6.70	10.52 ± 6.682	11.87 ± 6.72	0.302^a^
Total tic score	28.23 ± 8.86	26.79 ± 8.30	29.61 ± 9.23	0.101^a^
Comorbidities, n (%) (previously diagnosed)				
No comorbidities	50 (47.2%)	25 (48.1%)	25 (46.3%)	0.854^b^
ADHD	33 (31.1%)	15 (28.8%)	18 (33.3%)	0.618^b^
OCD	3 (2.8%)	2 (3.8%)	1 (1.9%)	0.614^c^
ASD	15 (14.2%)	5 (9.6%)	10 (18.5%)	0.189^b^
Anxiety disorder	6 (5.7%)	4 (7.7%)	2 (3.7%)	0.433^c^
Other	18 (17.0%)	10 (19.2%)	8 (14.8%)	0.545^b^
Comorbidities, n (%) (ADIS interview at baseline)				
No comorbidities	30 (28.3%)	19 (36.5%)	11 (20.4%)	0.065^b^
ADHD	59 (55.7%)	25 (48.1%)	34 (63.0%)	0.123^b^
OCD	18 (17.1%)	9 (17.3%)	9 (17.0%)	0.965^b^
Anxiety	35 (33.0%)	13 (25.0%)	22 (40.7%)	0.085^b^
Other	18 (17.0%)	9 (17.3%)	9 (16.7%)	0.930^b^
Previous treatment for tics or other psychiatric disorders, n (%)	60 (56.6%)	25 (48.1%)	35 (64.8%)	0.082^b^
Current treatment for tics or other psychiatric disorders (excl. ERP/HRT), n (%)	40 (38.1%)	17 (33.3%)	23 (42.6%)	0.329^b^
Occurrence of tics in the family, n (%)	47 (44.8%)	23 (45.1%)	24 (44.4%)	0.946^b^

Data obtained from intake interviews, unless otherwise indicated; numbers and percentages within randomisation groups

*SD* standard deviation, *ADHD* attention deficit hyperactivity disorder, *ASD* autism spectrum disorder, *OCD* obsessive–compulsive disorder, *ISCO-08* international standard classification of occupations, *YGTSS* Yale Global Tic Severity Scale

^a^T-test

^b^Chi-square test

^c^Fisher’s exact test

### Numbers analysed

In total, 106 participants were randomised to either the direct treatment condition (n = 52) or the waiting list control group (n = 54).

### Protocol adherence

Treatment integrity was high; a mean of 93% of the required elements per component was performed in accordance with the protocol (92% for ERP-sessions).

### Outcomes and estimation

Child and parental outcomes are summarised in Table 3. Data on all variables were normally distributed at baseline.

**Table 3:** Primary and Secondary Outcomes for Treatment Conditions on Three Timepoints

	TYT (n = 52)			WLCG (n = 54)				
	T1 Mean (± SD), n	T2 Mean (± SD), n	T3 Mean (± SD), n	T1 Mean (± SD), n	T2 Mean (± SD), n	T3 Mean (± SD), n	*p* value T1–T2	*p *value T1–T3
Tic severity (YGTSS total tic score (motor + vocal)	26.79 (± 8.30), 52	23.65 (± 7.85), 51	21.43 (± 8.12), 49	29.61 (± 9.23), 54	27.93 (± 10.41), 54	26.76 (± 10.68), 54	0.217	0.063
Motor tics (YGTSS total motor tic score)	16.26 (± 4.30), 52	14.80 (± 3.99), 51	13.30 (± 4.39), 49	17.74 (± 4.18), 54	16.63 (± 4.99), 54	15.76 (± 5.70), 54	0.601	0.183
Vocal tics (YGTSS total vocal tic score)	10.52 (± 6.68), 52	8.84 (± 6.39), 51	8.22 (± 6.40), 49	11.87 (± 6.72), 54	11.30 (± 6.97), 54	11.00 (± 6.99), 54	0.187	0.118
Tic-related impairment (YGTSS tic-related impairment score)	24.04 (± 11.59), 52	19.41 (± 13.18), 51	14.29 (± 11.18), 49	27.41 (± 12.16), 54	25.56 (± 12.24), 54	23.70 (± 13.36), 54	0.266	0.007*
Global tic severity (YGTSS global tic severity score)	50.83 (± 17.61), 52	43.06 (± 19.25), 51	36.20 (± 17.68), 49	57.02 (± 18.48), 54	53.48 (± 19.84), 54	51.50 (± 22.05), 54	0.182	0.001*
Quality of life – total problem score (C&A-GTS-QOL)	27.42 (± 17.96), 50	23.08 (± 18.78), 49	21.29 (± 16.55), 49	31.94 (± 20.06), 54	29.83 (± 19.72), 54	32.30 (± 22.04), 54	0.599	0.030*
Life satisfaction (C&A-GTS-QOL-VAS)^a^	72.89 (± 21.33), 47	77.30 (± 19.04), 46	79.29 (± 19.24), 45	74.89 (± 21.13), 53	73.09 (± 23.57), 53	74.29 (± 22.40), 52	0.047*	0.014*
Quality of life – cognitive problems (C&A-GTS-QOL-COGN)	5.36 (± 3.66), 50	4.75 (± 3.55), 48	4.28 (± 3.57), 47	6.07 (± 3.71), 54	5.37 (± 3.94), 54	6.00 (± 3.88), 53	0.696	0.166
Quality of life – physical problems (C&A-GTS-QOL-PHYS)	7.96 (± 5.62), 50	6.31 (± 5.35), 49	5.33 (± 4.49), 49	8.70 (± 5.85), 54	7.94 (± 5.62), 54	8.70 (± 6.05), 54	0.439	0.010*
Quality of life – obsessive/compulsive problems (C&A-GTS-QOL-OC)	4.24 (± 4.35), 50	3.47 (± 3.85), 49	3.43 (± 3.66), 49	4.69 (± 4.60), 52	4.55 (± 4.11), 53	5.50 (± 4.69), 54	0.688	0.071
Quality of life – psychological problems (C&A-GTS-QOL-PSYCH)	9.86 (± 7.73), 50	8.83 (± 8.68), 49	8.43 (± 7.96), 49	12.65 (± 9.71), 54	12.06 (± 9.10), 54	12.20 (± 10.38), 54	0.835	0.627
Tic-related cognitions (Beliefs about Tics, BATS)	42.24 (± 10.36), 51	38.94 (± 9.04), 49	–	47.46 (± 12.46), 54	46.04 (± 11.92), 54	–	0.420	
Emotional/behavioural functioning (CBCL6-18, total problem score)	41.80 (± 26.22), 51	–	33.06 (± 22.98), 50	43.36 (± 22.85), 47	–	42.70 (± 20.93), 53	–	0.014*
CBCL6-18, internalising problems	12.59 (± 9.34), 51	–	9.80 (± 9.68), 50	13.04 (± 9.36), 47	–	12.81 (± 8.47), 53		0.013*
CBCL6-18, externalising problems	8.31 (± 7.31), 51	–	6.30 (± 6.75), 50	8.23 (± 7.00), 47	–	8.25 (± 6.91), 53		0.183
Stress of parenting (Parenting stress index)	54.22 (± 13.57), 51	52.82 (± 12.58), 51	–	55.00 (± 14.93), 53	54.85 (± 14.76), 53	–	0.224	
Care-related quality of life in parents (CarerQOL)	85.45 (± 15.37), 52	86.10 (± 14.11), 51	–	83.45 (± 13.36), 52	83.57 (± 13.77), 53	–	0.865	
Family functioning (FAD)	41.02 (± 4.49), 51	41.00 (± 5.59), 51	––	40.74 (± 5.75), 53	41.30 (± 5.79), 53	–	0.359	

*YGTSS* Yale Global Tic Severity Scale, *C&A-GTS-QOL* Gilles de la Tourette Syndrome-Quality of Life Scale, *GTS-QOL-VAS* visual analogue scale for life satisfaction, *GTS-QOL-COGN* cognitive subscale, *GTS-QOL-PHYS* physical/activities of daily living subscale, *GTS-QOL-OC* obsessive–compulsive subscale, *GTS-QOL-PSYCH* psychological subscale, *BATS* Beliefs About Tics Scale, *CBCL6-18* Child Behaviour Checklist, *CarerQol* Care-related quality of life in informal caregivers questionnaire, *FAD* Family Assessment Device

^*^Significant findings (p < 0.05)

^a^Worse conditions indicated by lower scores

#### Tic severity (primary outcome)

**Fig. 2:**
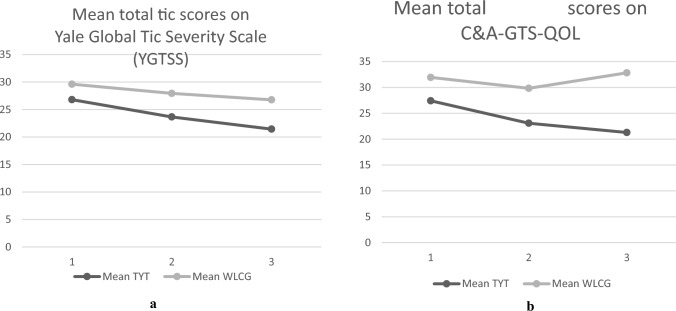
Mean Total Scores on the YGTSS (Total Tic Score) (**a**) and C&A-GTS-QOL (Total Problem Score) (**b**) on three time points (pre-treatment, post-treatment and 3-month follow-up). **a** Mean Total Scores on the YGTSS (Total Tic Score). **b** Mean Total Scores on the C&A-GTS-QOL (Total Problem Score). *YGTSS* Yale Global Tic Severity Scale, *C&A-GTS-QOL* Gilles de la Tourette Syndrome-Quality of Life Scale, *TYT* direct treatment group; *WLCG* waiting list control group

In the TYT group, the mean YGTSS total tic score decreased from T1 to T2 with 3.14 points (11.7%; 95% CI – 4.65 to – 1.27) and to T3 with 5.36 points (20%; 95% CI – 6.93 to – 3.11). This decrease showed no significant difference with the WLCG, that improved with 1.68 points (5.7%; 95% CI – 3.01 to – 0.37) at T2 and 2.85 points (9.6%; 95% CI – 4.51 to – 1.20) at T3 (see Fig. 2). At 6-months follow-up (T4), the WLCG had also received the treatment. Therefore, this group could no longer be handled as a control condition and between group differences over time could no longer be analysed for significance at T4. In the TYT group, the decreasing trend in tic severity continued to 6-months follow-up. The mean total tic severity score on T4 further decreased to 19.59 (– 7.2 points, 26.9% compared to T1). The WLCG was treated after T3 and showed a decrease between T3 and T4 in YGTSS total tic score (– 4.09, 15.3%). In the TYT group, the number of children with a positive response (reduced tic severity of  ≥ 25%) increased from 10 (19.6%) at T2 and 13 (26.5%) (of which 6 of the responders at T2) at T3 to 20 positive responders (39.2%) at T4 (of which 7 of the responders at T2). See Appendix 3 for a visualisation. In the WLCG, 7 children (13.0%) showed spontaneous reduced tic severity of  ≥ 25% at T2, 12 children (22.2%) at T3 and, after receiving treatment, 24 children (44.4%) showed a positive response at T4. Positive response rates did not significantly differ between the conditions (T2: χ^2^ (1, *N* = 105) = 0.853, *p* = 0.356; T3: χ^2^ (1, *N* = 103) = 0.259, *p* = 0.611; T4: χ^2^ (1, *N* = 105) = 0.295, *p* = 0.587). As there was only a single subject missing on the key outcome (TYT, at T2), no additional technique such as weighted generalised estimating equations (WGEEs) was used to handle missing data. Scores on the revised version YGTSS-R led to similar results, see Appendix 4.

Furthermore no significant group differences were found as to the mean decrease (T1–T2–T3) in motor tics and vocal tics separately.

#### Secondary outcomes

Tic-related impairment decreased from T1 to T2 with 4.63 points (19.3%; 95% CI – 8.54 to – 0.48) for TYT and 1.85 (6.7%; 95% CI – 4.76 to 1.06) for WLCG. At T3, the improvement (T1–T3) was 9.75 points (40.6%; 95% CI – 13.03 to – 6.56) compared to 3.71 in WLCG (13.5%; 95% CI – 7.03 to – 0.38), showing a significant superior effect of TYT (SMD = – 4.73). At T4, tic-related impairment in TYT decreased further to 12.55 (– 11.49, 47.8% compared to T1). The WLCG, treated after T3, showed a decrease of 6.11 points (25.8%).

The difference in change over time between the TYT condition and WLCG on quality of life was not statistically significant at T2 but did reach significance at T3 (SMD = – 0.283). Mean scores on the total problem score decreased in the TYT group with 4.34 points (15.8%; 95% CI – 7.74 to 1.28) from T1 to T2 and 6.13 (22.4%; 95% CI – 8.34 to – 1.53) from T1 to T3, while the scores of the WLCG remained relatively stable, showing a decrease of 2.11 points (6.6%; 95% CI – 4.87 to 0.64) from T1 to T2 and an increase to 0.36 points (1.1%; 95% CI – 3.58 to 4.29) from T1 to T3 (see Table 3). At T4, the total problem score in TYT further improved to 20.31 (– 7.11, 25.9% compared to T1). The WLCG showed a post-treatment decrease of 4.05 points (12.5%).

Significant differences in change were found on the life satisfaction score (0–100), both from T1 to T2 (SMD = 0.289), as well as from T1 to T3 (SMD = 0.289). Life satisfaction improved in the TYT group; mean scores increased with 4.41 points (6.1%; 95% CI – 0.62 to 6.90) from T1 to T2 and 6.4 points (8.8%; 95% CI 1.39–8.42) from T1 to T3, whereas in the WLCG this score decreased 1.8 points (2.4%; 95% CI – 5.53 to 1.94) from T1 to T2 and 0.6 points (0.1%; 95% CI – 4.45 to 2.84) between T1 and T3.

From T1 to T2, changes in tic-related cognitions, family functioning, including parenting stress and care related quality of life in parents, showed no significant differences over time between TYT and WLCG. Emotional and behavioural functioning, measured at T1 and T3 (Child Behaviour Checklist) showed a significant difference in change over time between TYT and WLCG with superior results for TYT (SMD = – 0.240). The total problem score decreased with 8.74 points (20.9%; 95% CI – 12.66 to 4.81) in the TYT group, whereas in the WLCG the decrease was 0.66 points (1.5%; 95% CI – 5.77 to 3.65). These improvements were mainly attributable to internalising problems, somatic complaints and problems, affective problems, ADHD problems and stress-related problems as indicated by significant differential changes on the CBCL-subscores.

#### Treatment satisfaction and adherence

Overall, participants in both conditions were satisfied about Tackle your Tics, after they had followed the treatment, see Appendix 5. Treatment satisfaction scores of the children showed mean scores of 3.72 (TYT 3.83; WLCG 3.61). For 95.8% of the children in the TYT group and 80% of the WLCG children, the mean score indicated a favourable treatment satisfaction. Parents’ mean score was 3.86 (TYT 3.96; WLCG 3.76) on a 5-point scale. For 98% of the parents in the TYT group and 86.3% of the WLCG parents, the mean score indicated a favourable treatment satisfaction. Mean SRS-scores of both groups were high (> 34) on a 0–40 scale. Children’s reports regarding homework adherence after the first three days of therapy was 3.70 (TYT 3.69; WLCG 3.72) on a 5-point scale (1 = not at all; 5 = a lot) and on parent reports regarding practicing together with their child 3.34 (TYT 3.22; WLCG 3.45) and 3.50 (TYT 3.46; WLCG 3.55) regarding the child’s practicing.

#### Predictors

Tic severity at baseline (total tic score, motor and vocal tic scores) significantly predicted differences in change of total tic severity over time (T1–T2–T3), indicating that higher pre-treatment tic scores predicted more improvement over time in the TYT compared to the WLCG condition. As for comorbidities, obsessive–compulsive disorder (n = 18; 9 TYT; 9 WLCG) predicted a differential change over time at T3, indicating more improvement of tic severity for TYT compared to WLCG. Premonitory urge severity, age, sex and family functioning did not significantly predict differential changes in tic severity, nor did externalising disorders, anxiety disorder, affective disorders, or posttraumatic stress disorder (see Table 4).

**Table 4:** Possible predictors of differential change over time in YGTSS Total Tic Score, on T2 and T3

Possible predictive factor	*p* value T2	*p* value T3
Tic severity at baseline (YGTSS total tic score)	0.010*	0.002*
Motor tics (YGTSS motor tic score)	0.132	0.034*
Vocal tics (YGTSS vocal tic score)	0.008*	0.003*
Premonitory urge severity (PUTS)	0.814	0.333
Age	0.263	0.190
Sex	0.820	0.747
Gender	0.220	0.512
Family functioning (FAD)	0.784	0.952
Comorbidity: any diagnosis (ADIS)	0.365	0.265
Externalising disorder (ADIS)	0.065	0.919
Obsessive–compulsive disorder (ADIS)	0.513	0.001*
Anxiety (ADIS)	0.063	0.250
Dysthymia (ADIS)	0.596	0.093
Depression (ADIS)	NA^a^	NA^a^
Post-traumatic stress disorder (ADIS)	0.699	0.232
Other (ADIS)	0.201	0.214

*YGTSS* Yale Global Tic Severity Scale, *PUTS* Premonitory Urges for Tics Scale, *FAD* Family Assessment Device, *ADIS* Anxiety Disorders Interview Schedule

^*^Significant findings (p < .05)

^a^No diagnosis in sample

## Discussion

Directly after treatment we did not find significant differences between TYT and WLCG on tic severity and secondary outcomes. The lack of effect on tic severity was unexpected, because previous studies into group formats as well as supportive programmes for tic disorders showed positive effects compared to control conditions. However, at 6 months post treatment the TYT group showed a decrease in total tic score of 7.2 points (26.9%) and 20 children (39.2%) were rated as responders. This decrease is comparable to results of previous treatment studies [16, 35, 36]. Long-term improvement of tic-related impairment, quality of life and emotional and behavioural functioning (especially internalising problems) are in line with those of other treatments (e.g., [13, 14, 37]). Higher tic severity at baseline and obsessive–compulsive disorder predicted improvements over time on tic severity, which is also found in other studies [38, 39]. Our results indicate that Tackle your Tics does not lead to faster tic reduction, but that children can benefit from this brief intensive treatment, provided in four consecutive days. Benefits were especially related to enhanced quality of life, tic-related impairment and behavioural and emotional functioning.

Both at short and longer term, offering treatment in this brief intensive format, together with a ‘total package’ of supportive elements did not lead to the expected optimisation of the main treatment outcome: tic severity. There are several possible explanations for not finding superior effect of TYT regarding tic severity compared to WLCG. First, our key time point was planned directly after the brief treatment; one week after the fourth ‘booster day’. This offered limited time and opportunity to practice ERP-exercises at home and to generalise newly acquired skills to different situations. Generalisation was earlier hypothesised to be important by Blount and colleagues [17]. From the pilot study we learned that this intensive format was feasible and offered advantages for children and their families. However, intensive treatment may also have been associated with time pressure, a continued focus on and awareness of tics, an accelerated process of acceptance, change of environment (hotel stays) and fatigue. This could have affected the direct post treatment outcomes. It is possible that a more sequential format, or additional booster sessions are needed to enhance generalisation and treatment effects. However, our follow-up results indicate that the skills learned in a brief, intensive treatment need a longer time interval to achieve relevant tic reductions.

Second, the participants in the waiting list condition also completed interviews and questionnaires. This may have increased insight and awareness of tics and related problems, possibly having positive impact on their symptoms. Spontaneous improvement due to reassurance of getting treatment is well-known [35].

Furthermore, having gained more control over the tics was most often mentioned in the treatment satisfaction forms by parents (38%) as helpful factor. It is known that YGTSS scores are not very sensitive to detect phenomena related to behavioural treatment, such as the ability to suppress tics [40]. Scores on the frequency dimension are known to be skewed and often high [31]. In participants with improved control after treatment, a change in tic-free intervals of only a few seconds to a few minutes can be experienced as a substantial improvement in tic severity, that is not reflected in the frequency score.

Finally, as a unique element of the programme, we offered workshops led by experts by experience. Acceptance of having tics was an important topic in these workshops. The ability to suppress tics when needed and normalisation of tics in a group setting may unintendedly have reduced the motivation to practice the ERP-exercises at home. This could have been hindering tic reduction. Children may have thought: “When my tics are okay and in control, why work hard to get rid of them?” This raises the question of what the definition of a successful treatment outcome is: tic reduction or no need for tic reduction? Favourable treatment satisfaction scores and low drop-out indicate this is a valuable and acceptable new treatment format for youngsters with chronic tics or Tourette syndrome.

### Strengths and limitations

This study is the first randomised controlled trial with a large sample size studying the effectiveness of a brief, intensive group ERP-treatment for youth with tic disorders. We used a broad age range and validated assessment instruments, with a multi-informant approach. Measurements were conducted online in the home environment, which provides a more accurate impression of the course of tics and other outcomes compared to a clinical setting.

The clinical profit included the training of 24 therapists by experienced therapists. This expanded future access to local behavioural treatment for tic disorders. Finally, the results, both positive and negative, are relevant for the development of various new modalities to optimise and broaden the outcomes of behavioural treatment.

Limitations of this study include the lack of an active control group. For this new treatment we chose to determine efficacy in relation to a waiting list condition. For an active control group, we would have needed an even larger sample size and more available experienced therapists, which was not feasible. However, the waiting list group controlled for several confounding factors such as expectancy effects, possible seasonal effects and the natural waxing and waning course of tics in time. The fact that tics fluctuate over time, however, could also have caused random improvement or worsening, making it more difficult to find group differences. No correction was applied for multiple testing (such as the Bonferroni correction which can be considered as too conservative). Therefore, these results have to be interpreted with caution, because of multiple comparisons in a relatively small sample.

Finally, this study was conducted during the COVID-19 pandemic. Although no major adaptations to the programme (e.g. online sessions) were needed for the TYT condition during the 4 treatment days, effects of minor adjustments (e.g. 1.5 m distance, face masks) and family stress as a result of the COVID-19 pandemic and lockdowns may have influenced treatment results, such as outcomes on parenting stress and caregiver burden.

### Future directions

Based on our findings, we do not advise this brief, intensive group treatment as a ‘quick fix’ for reducing tics. However, we do recommend Tackle your Tics for children and adolescents to improve their quality of life on long term, to better cope with their tics and behavioural and emotional problems and to feel less impaired. Patient organisations have been emphasising the importance of quality of life over tic reduction for a long time. It could help many children and their families if science and clinical practice also set quality of life an important goal in the development of new modalities to broaden treatment for tic disorders. Future research could specifically examine the surplus value of experiential knowledge in improving quality of life, for example in studies comparing interventions with and without this element. Our findings on predictive factors stress the need to provide different treatment options to meet each patients individual needs.

## Conclusions

Our results directly after treatment indicate no superior effect of a brief, intensive ERP group treatment for children and adolescents with chronic tics on tic reduction, compared to a waiting list condition. However, at 3 months follow-up, Tackle your Tics was effective in improving tic-related impairment, quality of life and behavioural/emotional functioning, providing a positive treatment satisfaction.

## Supplementary Information

Below is the link to the electronic supplementary material.

Supplementary file1 (DOCX 28 KB)

Supplementary file2 (DOCX 23 KB)

Supplementary file3 (DOCX 37 KB)

Supplementary file4 (DOCX 23 KB)

Supplementary file5 (DOCX 42 KB)

## Data Availability

Data are available upon reasonable request. Considering the fact that for this project we have gathered highly sensitive and privacy protected data (protection legislated by Dutch law), we can share relevant supporting documents, in case of relevant research questions. For this study, the following study information will be available: metadata about the study: the study protocol, statistical analysis plan, data management plan, metadata schema and a global description of the intervention protocols (as described in our trial design paper by Heijerman-Holtgrefe et al. [22], metadata about the data: documentation on study procedures, a data dictionary and syntaxes.

## Footnotes

1 We use the term Tourette Syndrome rather than Tourette’s Disorder as stated in the Diagnostic and Statistical Manual of Mental Disorders, 5th edition (DSM-5), because this better reflects the complexity across the full spectrum of tics and comorbidities, and is preferred by the patient community.
