# Supplementary material for: Tackle your Tics, a brief intensive group-based exposure treatment for young people with tics: results of a randomised controlled trial
Source: Eur Child Adolesc Psychiatry. 2024 Apr 4;33(11):3805–18. doi: 10.1007/s00787-024-02410-0 (PMC11588865; doi:10.1007/s00787-024-02410-0)
Supplement: Supplementary file 1 — Supplementary file1 (DOCX 28 KB) [file 787_2024_2410_MOESM1_ESM.docx]

**Appendix 1**

*Tackle your Tics Therapy Programme for Children and Adolescents*

|  | Day 1 | Day 2 | Day 3 | Day 4 booster day (after 1 week) | Get together afternoon  (after 1 month) | |
| --- | --- | --- | --- | --- | --- | --- |
| 9:30-10:00 | Welcome and  acquaintance game (all participants) |  |  |  |  | |
| 10:00-11:00 | Psychoeducation or therapy session (subgroups) | Psychoeducation or therapy session  (subgroups) | Psychoeducation or therapy session  (subgroups) | Psychoeducation or therapy session  (subgroups) |  | |
| 11:00-11:10 | Short break (all participants) | Short break (all participants) | Short break (all participants) | Short break (all participants) |  | |
| 11:10-12:10 | Psychoeducation or therapy session  (subgroups) | Psychoeducation or therapy session  (subgroups) | Psychoeducation or therapy session  (subgroups) | Psychoeducation or therapy session  (subgroups) |  | |
| 12:10-12:40 | Lunch break (all participants) | Lunch break (all participants) | Lunch break (all participants) | Lunch break (all participants) |  | |
| 12:40-13:40 | Workshop coping strategies or therapy session (subgroups) | Workshop coping strategies or therapy session (subgroups) | Workshop coping strategies or therapy session (subgroups) | Workshop coping strategies or therapy session (subgroups) |  | |
| 13:40-14:10 | Playtime  (all participants) | Playtime  (all participants) | Playtime  (all participants) | Playtime  (all participants) |  | |
| 14:10-15:10 | Workshop coping strategies or therapy session (subgroups) | Workshop coping strategies or therapy session (subgroups) | Workshop coping strategies or therapy session (subgroups) | Workshop coping strategies or therapy session (subgroups) | 14:00- 14:30 | Get together  (all participants): welcome |
| 15:10-15:25 | Relaxation therapy (all participants) | Relaxation therapy (all participants) | Relaxation therapy (all participants) | Relaxation therapy (all participants) | 14:30- 15:30 | Evaluation  (all participants) |
| 15:25-15:40 | group therapy session  (all participants) | group therapy session  (all participants) | group therapy session  (all participants) | group therapy session  (all participants) | 15:30- 15:45 | Break/playtime |
| 15:40-15:55 | Short evaluation (all participants) | Short evaluation (all participants) | Short evaluation (all participants) | Short evaluation (all participants) | 15:45- 16:45 | Workshop  (all participants) |
| 15:55-16:30 | Feedback:  therapist with parents and participant | Feedback:  therapist with parents and participant | Feedback:  therapist with parents and participant | Feedback:  therapist with parents and participant | 16:45- 17:00 | Get together  (all participants): closing |

For all groups (direct treatment and waiting list condition), three online parent meetings were organised at pre-treatment, post-treatment and after the ‘get together afternoon’. Psychoeducation and workshops were offered in subgroups of 3-5 children, parallel to the therapy sessions of the other participants (except for small groups that consisted of 4 or 5 children).
