# Supplementary material for: Tackle your Tics, a brief intensive group-based exposure treatment for young people with tics: results of a randomised controlled trial
Source: Eur Child Adolesc Psychiatry. 2024 Apr 4;33(11):3805–18. doi: 10.1007/s00787-024-02410-0 (PMC11588865; doi:10.1007/s00787-024-02410-0)
Supplement: Supplementary file 2 — Supplementary file2 (DOCX 23 KB) [file 787_2024_2410_MOESM2_ESM.docx]

**Appendix 2**

*Participant flow details*

See Figure 1 for a schedule of the participation flow

In total, 164 families were assessed for eligibility; 31 children did not meet the inclusion criteria, because they either were not motivated for treatment (n=10), not bothered by their tics (n=2) or both (n=4), were not diagnosed with TS or CTD or the tics started less than 12 months ago (n=6), had received behavioural treatment for tics in the past 12 months (n=3), had minimal tic severity (n=3), were older than 17 years (n=1), had no mastery of the Dutch language (n=1) or had an IQ below 75 (n=1). Of the 133 eligible families, 8 families preferred another treatment for tics or co-occurring problems, 8 families had other, practical (n=4) or emotional (n=4) reasons that prevented them from participating and 9 families did not reply after the initial invitation.

Included children (n=108) and eligible non-participants (n=25) did not differ as to children’s age (participants: M=12.31 years, SD=2.18; non-participants: M=12.47, SD=2.17; t(131)=-.091; p=.76), sex (male: 66.7% (participants) and 56.0% (non-participants); χ^2^ (1, N=133)=1.01, p=.32) and residential area (Noord-Holland: 39.8% (participants) and 24.0% (non-participants); Fisher’s exact test, p=.46). After randomisation of 108 children and adolescents, two families dropped out before the first assessment, because they were not satisfied with the dates of the treatment. Because of legitimate, pressing reasons (e.g., Covid-19, school final exams), 16 participants (8%) were unable to attend their allocated TYT group. For 9 of them it was possible to move on to the next TYT group. For 7 participants this was not possible (e.g., because of allowed group size) and manual allocation to another treatment condition was necessary. Children and adolescents and their parents were recruited from July 2020 to May 2022. The researcher (AH) became unblinded for a total of 7 participants, e.g., by participants who, despite clear instructions not to tell, accidentally mentioned their condition during the interviews.

In the TYT condition, one participant dropped out after day 3 of treatment (due to lack of motivation), and two families missed more than two hours of the first two parent meetings. There was one adverse event beween T2 and T3, concerning a participant in the TYT condition, who was voluntarily admitted to a clinic for other, not tic-related problems. In the WLCG that received treatment after 3 months, 3 participants dropped out (2 due to minimal/absence of tics, 1 due to poor group functioning). Between T1 and T2, five participants allocated to WLCG, reported a change in medication (3 raised, 2 lowered). Between T2 and T3, six participants reported a change in medication (2 TYT, raised medication; 4 WLCG, 2 raised, 2 lowered). In the WLCG, for 8 children (7 of one treatment group) online participation was needed to attend the fourth day of treatment (including 135 minutes of ERP-sessions), due to quarantine or Covid-19. In TYT, no online sessions were needed during the 4 treatment days. Sensitivity analyses considering protocol deviations resulted in the same conclusions.
