# Supplementary material for: Tackle your Tics, a brief intensive group-based exposure treatment for young people with tics: results of a randomised controlled trial
Source: Eur Child Adolesc Psychiatry. 2024 Apr 4;33(11):3805–18. doi: 10.1007/s00787-024-02410-0 (PMC11588865; doi:10.1007/s00787-024-02410-0)
Supplement: Supplementary file 3 — Supplementary file3 (DOCX 37 KB) [file 787_2024_2410_MOESM3_ESM.docx]

**Appendix 3**

*Tic severity course at all four time points for quick responders (25% or more reduction on the YGTSS total tic score between T1 and T2) in the TYT condition.*
