# Supplementary material for: Tackle your Tics, a brief intensive group-based exposure treatment for young people with tics: results of a randomised controlled trial
Source: Eur Child Adolesc Psychiatry. 2024 Apr 4;33(11):3805–18. doi: 10.1007/s00787-024-02410-0 (PMC11588865; doi:10.1007/s00787-024-02410-0)
Supplement: Supplementary file 4 — Supplementary file4 (DOCX 23 KB) [file 787_2024_2410_MOESM4_ESM.docx]

**Appendix 4**

| YGTSS and YGTSS-R comparison | | | | | |
| --- | --- | --- | --- | --- | --- |
|  | TYT (n=52) | | WLCG (n=54) | | |
|  | T1 | T2 | T1 | T2 | *p*-value |
|  | Mean (±SD), n | Mean (±SD), n | Mean (±SD), n | Mean (±SD), n |  |
| Tic severity  (YGTSS total tic score (motoric + vocal) | 26.79 (±8.30), 52 | 23.65 (±7.85), 51 | 29.61 (±9.23), 54 | 27.93 (±10.41), 54 | .217 |
| Tic severity  (YGTSS-R total tic score (motoric + vocal) | 27.15 (±8.40), 52 | 24.06 (±7.91), 51 | 30.35 (±9.14) | 28.69 (±10.13), 54 | .236 |
| Motor tics (YGTSS total motor tic score) | 16.26 (±4.30), 52 | 14.80 (±3.99), 51 | 17.74 (±4.18), 54 | 16.63 (±4.99), 54 | .601 |
| Motor tics (YGTSS-R total motor tic score | 16.50 (±4.33), 52 | 15.12 (±4.06), 51 | 18.20 (±4.09), 54 | 17.13 (±4.86), 54 | .669 |
| Vocal tics (YGTSS total vocal tic score) | 10.52 (±6.68), 52 | 8.84 (±6.39), 51 | 11.87 (±6.72), 54 | 11.30 (±6.97), 54 | .187 |
| Vocal tics (YGTSS-R total vocal tic score) | 10.65 (±6.71), 52 | 8.94 (±6.34), 51 | 12.15 (±6.72), 54 | 11.56 (±6.97), 54 | .180 |
| Global tic severity (YGTSS global tic severity score) | 50.83 (±17.61), 52 | 43.06 (±19.25), 51 | 57.02 (±18.48), 54 | 53.48 (±19.84), 54 | .182 |
| Global tic severity (YGTSS-R global tic severity score) | 51.19 (±17.66), 52 | 43.47 (±19.45), 51 | 57.76 (±18.29), 54 | 54.24 (±19.57), 54 | .187 |
