# Supplementary material for: Tackle your Tics, a brief intensive group-based exposure treatment for young people with tics: results of a randomised controlled trial
Source: Eur Child Adolesc Psychiatry. 2024 Apr 4;33(11):3805–18. doi: 10.1007/s00787-024-02410-0 (PMC11588865; doi:10.1007/s00787-024-02410-0)
Supplement: Supplementary file 5 — Supplementary file5 (DOCX 42 KB) [file 787_2024_2410_MOESM5_ESM.docx]

**Appendix 5**

*Treatment satisfaction and adherence scores post treatment for TYT group (at T2 and WLCG (at T4)*

|  | | | | |  |  |  |
| --- | --- | --- | --- | --- | --- | --- | --- |
|  | TYT (n=48) | | | | WLCG (n=51) | | |
| 1. Child questionnaires | | **mean** | **range** | **SD** | **mean** | **range** | **SD** |
| Questions on a 1-5 points scale*: | |  |  |  |  |  |  |
| Did the training help you with your problems? | | 3.98 | 2-5 | .699 | 3.69 | 1-5 | 1.104 |
| You have practiced and learned a lot in a short time. Was that feasible? | | 4.00 | 2-5 | .715 | 3.67 | 1-5 | .993 |
| What did you think of the number of days?** | | 4.17 | 1-5 | 1.155 | 4.14 | 1-5 | 1.217 |
| Was the training as you expected? | | 3.77 | 1-5 | .973 | 3.22 | 1-5 | 1.119 |
| Did you think everything was easy to understand? | | 4.27 | 2-5 | .707 | 4.35 | 3-5 | .559 |
| Has the therapy helped you? (practicing with the therapists) | | 4.00 | 3-5 | .619 | 3.80 | 1-5 | 1.217 |
| Have your tics been reduced? | | 3.19 | 1-5 | 1.024 | 3.29 | 1-5 | 1.301 |
| Did the education in the morning help you? (about tics, urges, triggers and such) | | 3.69 | 1-5 | .829 | 3.57 | 1-5 | 1.044 |
| Did the app (BT coach) help you practice? | | 2.67 | 1-5 | 1.226 | 2.22 | 1-5 | 1.301 |
| Did the relaxation exercises help you? (breathing exercises) | | 3.08 | 1-5 | 1.145 | 2.92 | 1-5 | 1.163 |
| What did you think of the lunch break and outdoor play time?** | | 4.58 | 3-5 | .821 | 4.41 | 1-5 | 1.080 |
| Did the workshop in the afternoon help you? (learning to deal positively with tics and other problems) | | 3.71 | 1-5 | .944 | 3.55 | 1-5 | 1.154 |
| Did it help you that you practiced together with other children or young people with tics? | | 4.23 | 1-5 | .928 | 3.75 | 1-5 | 1.214 |
| Would you recommend this training to other children with tics? | | 4.29 | 3-5 | .582 | 4.00 | 1-5 | 1.114 |
| Mean score | | 3.83 | 2.43-4.64 | .484 | 3.61 | 1-5 | .754 |
| Other questions | |  |  |  |  |  |  |
| What rating would you give the entire week? (1-10 scale) | | 8.21 | 5-10 | 1.110 | 7.57 | 3-10 | 1.754 |
| Would you rather have had the training/therapy on your own? (yes/no/don’t know) | | Yes:  6.3% | No: 87.5% | I don’t know: 6.3% | Yes: 8.0% | No: 80.0% | I don’t know: 12.0% |
| Adherence | | mean | range | SD | mean | range | SD |
| Did you practice after your first 3 training days?  (1-5 scale; 1=not at all; 2=not really; 3= a little; 4=yes; 5= a lot) | | 3.69 | 2-5 | .689 | 3.72 | 1-5 | 1.107 |

|  |  |  |  |  |  |  |
| --- | --- | --- | --- | --- | --- | --- |
|  | TYT (n=50) | | | WLCG (n=51) | | |
| 1. Parents questionnaire (n=50) | mean | range | SD | mean | range | SD |
| Questions about the child, on a 1-5 points scale*: |  |  |  |  |  |  |
| How helpful was this therapy for your child’s problems? | 3.84 | 1-5 | 1.127 | 3.67 | 1-5 | 1.244 |
| How much do you think your child's tics have been reduced? | 3.17 | 1-5 | 1.307 | 3.08 | 1-5 | 1.495 |
| Your child had a lot of therapy and other activities in a short period of time. Was this feasible for him or her? | 4.24 | 2-5 | .804 | 4.00 | 1-5 | .969 |
| What did you think of the number of days?** | 4.51 | 3-5 | .869 | 4.59 | 1-5 | .998 |
| Every morning the therapists educated about tics, 'tic alarms' that precede the tics, stimuli that trigger tics, etc. Do you think this explanation helped your child? | 4.19 | 2-5 | .741 | 3.82 | 1-5 | 1.014 |
| Your child has practiced with the therapists every day to recognize and pay attention to the 'tic alarms' just before a tic. Do you think those exercises helped your child? | 4.06 | 2-5 | .704 | 3.78 | 1-5 | 1.064 |
| Your child has also done exercises with the therapists to stop the tics for as long as possible. Do you think those exercises helped your child? | 4.09 | 2-5 | .717 | 3.73 | 1-5 | 1.168 |
| Has the contact and practicing with other children helped your child? | 4.02 | 1-5 | .956 | 3.90 | 1-5 | 1.118 |
| The 'BT-Coach' app was used in the therapy. Has this app helped you and your child practice? | 2.97 | 1-5 | 1.325 | 2.52 | 1-5 | 1.278 |
| Have the relaxation exercises helped your child? (breathing exercises) | 3.00 | 1-5 | .803 | 2.93 | 1-5 | 1.074 |
| How did your child feel about the lunch break and outdoor play time?** | 4.79 | 3-5 | .615 | 4.73 | 1-5 | .817 |
| Every afternoon, experts by experience gave a workshop on dealing positively with tics and other complaints. Have these workshops helped your child? | 3.93 | 2-5 | .721 | 3.94 | 1-5 | 1.071 |
| Did the therapy program meet your expectations? | 4.02 | 2-5 | .553 | 3.76 | 1-5 | .907 |
| Have you received sufficient explanation about the content of the therapy? | 3.96 | 3-5 | .348 | 3.84 | 2-5 | .505 |
| Was the therapy and the explanation you received about it easy to understand? | 4.06 | 4-5 | .242 | 4.06 | 4-5 | .238 |
| Have you been given enough tools to help your child practice, even after the therapy? | 3.76 | 2-5 | .591 | 3.69 | 1-5 | .836 |
| Have the parent meetings helped you? | 3.22 | 1-4 | .815 | 2.88 | 1-4 | .952 |
| Did the conversations with the therapist at the end of the day help you? (feedback) | 3.82 | 1-5 | .755 | 3.57 | 1-5 | .878 |
| In therapy, your child has learned techniques that help control and actually reduce the tics through practice. In addition, we discussed that tics are not wrong and are allowed. Did this message get across sufficiently? | 4.08 | 3-5 | .396 | 4.00 | 1-5 | .632 |
| How did you feel about the extent to which you were involved in the program as a parent?** | 4.76 | 3-5 | .657 | 4.40 | 1-5 | 1.010 |
| Are you satisfied with this form of treatment (4 days of intensive practice, combined with supportive and motivational activities)? | 4.08 | 3-5 | .566 | 3.94 | 2-5 | .705 |
| Would you recommend this intensive form to other parents of children with tics? | 4.22 | 3-5 | .545 | 4.04 | 1-5 | .871 |
| Mean score | 3.96 | 3.00 -4.68 | .355 | 3.76 | 1.81-4.68 | 6.094 |
| Other questions | Yes | No | I don’t know | Yes | No | I don’t know |
| Would you have preferred your child to receive the training individually? | 8% | 76% | 16% | 7.8% | 84.3% | 7.8% |
| Did you need any other information that was not discussed? | 18% | 82% | NA | 11.8% | 88.2% | NA |
| Do you need follow-up or aftercare? | 74% | 26% | NA | 38.0% | 62.0% | NA |
| Adherence | mean | range | SD | mean | range | SD |
| Did you practice with your child after the first 3 days of therapy?  (1-5 scale; 1=not at all; 2=not really; 3= a little; 4=yes; 5= a lot) | 3.22 | 1-5 | .996 | 3.45 | 1-5 | .966 |
| Has your child practiced after the first 3 days of therapy?  (1-5 scale; 1=not at all; 2=not really; 3= a little; 4=yes; 5= a lot) | 3.46 | 1-5 | .885 | 3.55 | 1-5 | .901 |
| Were you present at the parent meetings? | Yes: 94% | No: 6% | NA | Yes: 96.1% | No: 2.0% | NA |

|  |  |  |  |  |  |  |  |  | |
| --- | --- | --- | --- | --- | --- | --- | --- | --- | --- |
|  | TYT (n=48) | | | | WLCG (n=49) | | | |  |
| 1. ORS/SRS questionnaires | Day 1 | Day 2 | Day 3 | Day4 | Day 1 | Day 2 | Day 3 | Day4 | |
| Outcome rating scale (mean total score 0-40, SD):  How are you doing? | 31.50 (±5.47) | 33.14 (±5.12) | 33.95  (±4.68) | 34.70  (±4.10) | 31.65 (±5.94) | 32.28 (±7.35) | 33.59 (±5.68) | 34.32 (±4.66) | |

*5-point Likert scale questions (1=very negative/not helpful at all; 5=very positive/helpful);
**questions with other response categories that have been converted to a 5-point scale
(1=way too little/much; 3=a bit too little/much; 5=exactly right)
